# Higher baseline heart rate variability in CCHS patients with progestin-associated recovery of hypercapnic ventilatory response

**DOI:** 10.1186/s12931-023-02625-w

**Published:** 2024-02-09

**Authors:** Caroline Sevoz-Couche, Maxime Patout, Beny Charbit, Thomas Similowski, Christian Straus

**Affiliations:** 1 Sorbonne Université, UPMC Univ Paris 06, INSERM, UMRS1158 Neurophysiologie Respiratoire Expérimentale et Clinique, 75013 Paris, France; 2 Département R3S (Respiration, Réanimation, Réadaptation Respiratoire, Sommeil), Service des Pathologies du Sommeil, AP-HP, Groupe Hospitalier Universitaire APHP-Sorbonne Université, Hôpital Pitié-Salpêtrière, 75013 Paris, France; 3 Département R3S (Respiration, Réanimation, Réadaptation Respiratoire, Sommeil), Centre de Référence Constitutif Maladies Pulmonaires Rares de l’Adulte Orphalung, Hypoventilations Centrales, Syndrome d’Ondine, AP-HP, Groupe Hospitalier Universitaire APHP-Sorbonne Université, Hôpital Pitié-Salpêtrière, 75013 Paris, France; 4 Faculté de Médecine, EA 3801, Université de Reims Champagne Ardenne, 51095 Reims, France; 5 Anesthesia, Critical Care and Pain Medicine Department, CHU Reims, Hôpital Robert Debré, 51092 Paris, France; 6 Département R3S (Respiration, Réanimation, Réadaptation Respiratoire, Sommeil), AP-HP, Groupe Hospitalier Universitaire APHP-Sorbonne Université, Hôpital Pitié-Salpêtrière, 75013 Paris, France; 7 Département R3S (Respiration, Réanimation, Réadaptation Respiratoire, Sommeil), Service d’Explorations Fonctionnelles de la Respiration, de l’Exercice et de la Dyspnée, AP-HP, Groupe Hospitalier Universitaire APHP-Sorbonne Université, Hôpital Pitié-Salpêtrière, 75013 Paris, France

**Keywords:** Hypoventilation syndrome, Desogestrel, Autonomic nervous system, Heart rate variability

## Abstract

After a fortuitous observation of two cases of chemosensitivity recovery in women with congenital central hypoventilation syndrome (CCHS) who took desogestrel, we aimed to evaluate the ventilatory response to hypercapnia of five CCHS patients with or without treatment consisting of desogestrel (DESO) or levonorgestrel (LEVO). Only two patients became responsive to hypercapnia under treatment, according to their basal vagal heart rate variability. These results suggest that heart rate variability may be promising tool to discriminate patients susceptible to become responsive to hypercapnia under DESO-LEVO treatment.

*Clinical Trials Identifier* NCT01243697

## Introduction

In humans, heterozygous expansions of a normal 20-alanine repeat sequence in the PHOX2B gene (PARM mutation) cause an array of phenotypic defects. Affected patients typically suffer from central hypoventilation (hence the congenital central hypoventilation syndrome denomination, CCHS) that requires ventilatory assistance during sleep and is characterized by reduced or absent CO_2_-chemosensitivity [1]. Studies point at a developmental defect of PHOX2B-expressing CO_2_/H + sensitive neurons in the retrotrapezoid nucleus as the source of the chemosensitivity defect [2]. Among various vegetative anomalies, CCHS patients also exhibit cardiovascular autonomic dysregulation [3]. Building on the serendipitous description of two cases of CO_2_-chemosensitivity recovery in CCHS women receiving desogestrel, a progestin contraceptive [4], we conducted a clinical trial to examine the effects of desogestrel treatment on the ventilatory response to CO_2_ (RESPIRONDINE study, NCT01243697). We also examined the cardiac autonomic balance, keeping in mind that acute hypercapnia is associated with an increase in vagal tone in healthy subjects [5]. A preliminary form of this work was published as an abstract for a national congress, Les Journées de Recherche Respiratoire 2014 (Rev Mal Respir 2015: 32 (3), 338).

## Methods

Inclusion criteria were: CCHS pubescent females, carrying a PARM mutation and without contraindication to desogestrel treatment. Non-inclusion criteria were: contraindication to desogestrel treatment, estrogen-progestogen therapy for another purpose than contraception, pregnancy, non PARM mutation. Patients not previously under oral contraception (arm#1) were studied at baseline (“without treatment”), prescribed desogestrel 75 μg/day for 3 weeks to 3 months, and studied again (“with treatment”). Patients previously taking an oral contraception not comprising desogestrel nor levornorgestrel (arm#2) were asked to interrupt it for 4 months, after which they followed the same procedure as in arm#1. Patients previously taking a desogestrel or levonorgestrel based contraception (arm#3) were studied at baseline (“with treatment”) and then after 1 to 4 months of treatment discontinuation (“without treatment”).

The response to CO_2_ was studied using Read’s closed circuit rebreathing technique [6], starting with a 7%CO_2_—93%O_2_ gas mixture (Hyp’Air Compact +, Medisoft, Sorinnes-Dinant, Belgium), and characterized in terms of the slope of the minute ventilation (V’E)—end-expiratory end tidal CO_2_ partial pressure (PETCO2) expressed in L.min^−1^.mmHg^−1^. CO_2_ sensitivity was also assessed by the change in V’E (∆V’E) between steady-state air breathing (normoxia) and exposure to hyperoxic hypercapnia (5% CO_2_—95% O_2_). V’E was calculated as the mean V’E between the 5th and the 10th minute of gas mixture exposure. Electrocardiographic recordings (EKG) collected during steady-state air breathing and 5%CO_2_ hypercapnia were used to characterize autonomic cardiac regulation through an analysis of heart rate variability (HRV) using Poincaré plots [7], a type of recurrence plot where one takes a sequence of intervals and plots each interval against the following interval (R-R interval in the case cardiac studies). The dispersion across the line of identity (the “plot width”, SD1) reflects short-term variability (a marker of the vagal activity in the case of HRV). The length of the scattergram along the line of identity reflects long-term variability (SD2, a marker of the summation of sympathetic and parasympathetic activities in the case of HRV). The SD2/SD1 ratio is a marker of sympathetic activity [8].

Power spectra from R-R intervals were obtained by Fourier transformation (size 128, Hanning window, giving results with frequencies from 0 to 0.5 Hz and a final frequency resolution of 0.007 Hz). Low- (LF) and high-frequency (HF) powers were within the ranges of 0.04–0.15 Hz and 0.15–0.40 Hz, respectively [9]. LF (a reflection of the summation of sympathetic and parasympathetic activities [10, 11]) and HF (a marker of the vagal tone [12, 13]) powers (ms2/Hz) were calculated. The ratio between LF and HF bands (LF/HF, a marker of the sympathetic activity) were determined [14].

## Results

Five patients were included in the study (arm#1: #1: age 31, 20/26 alanine expansion; #2: age 30, 20/29. Arm#2: #3: age 24, 20/27. Arm#3: #4: age 22, 20/25; #5: age 24, 20–26). According to the CO_2_ rebreathing data, patients #1, #2 and #3 kept a near-abolished ventilatory response in the “with treatment” condition compared to the “without treatment” (0.26 vs 0.01 L.min^−1^.mmHg^−1^, 0.18 vs 0.18 L.min^−1^.mmHg^−1^_,_ and 0.22 vs 0.08 L.min^−1^.mmHg^−1^ respectively). A steady-state response to 5% CO_2_ (∆V’E) was present but low and not clearly sensitive to treatment (∆V’E: 0.57 vs 0.89 L.min^−1^, 1.35 vs 1.63 L.min^−1^ and 2.8 vs 1.5 L.min^−1^ with and without treatment, respectively). Patients #4 and #5 had a normal ventilatory response to rebreathing (1.76 and 2.55 L.min^−1^.mmHg^−1^, respectively) in the "with treatment condition". Of note, patient #4 was taking desogestrel for four years and patient #5 a combination of levonorgestrel and ethinylestradiol for almost three years at the time of inclusion (arm#3). Both of them had no ventilatory response to CO_2_ before taking oral contraception. The recovery of a response in patient #4 with desogestrel had previously been published [4]. In the current study, their CO_2_ response were still normal but lower in the “without treatment” condition, namely after 1 to 4 month washout (1.07 and 1.94 L.min^−1^.mmHg^−1^, respectively). In the steady state condition, ∆V’E was 2.42 L.min^−1^ for patient #4 with treatment and not available for technical reasons without treatment. For patient #5, ∆V’E was 4.27 L.min^−1^ and 0.56 L.min^−1^ with and without treatment respectively.

**Fig. 1:**
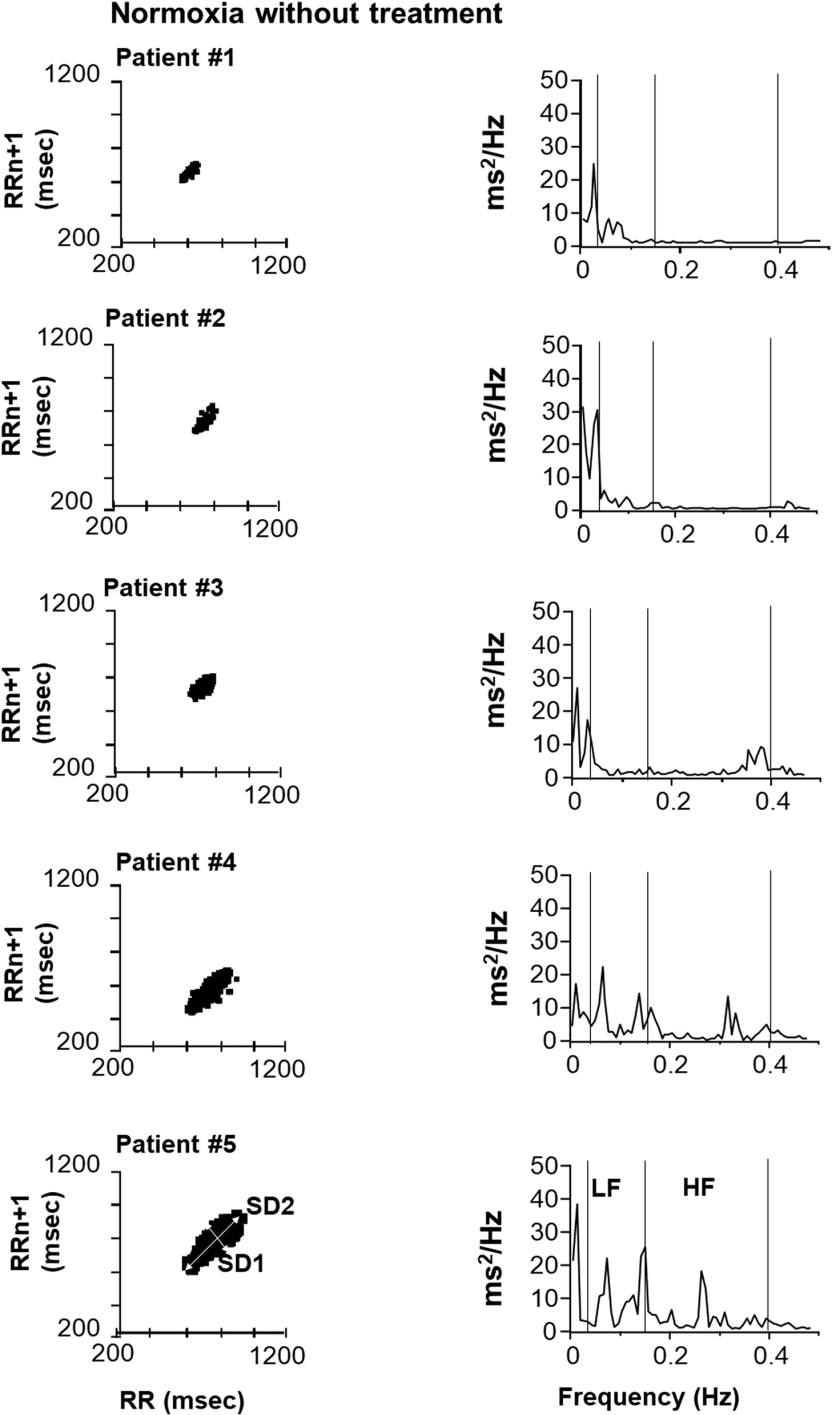
Qualitative beat-to-beat analysis of RR-intervals during steady state air breathing (normoxia) using Poincaré analysis and Frequential analysis. Note the higher dispersion of points in Poincare ellipses in Patients 4 and 5 (responsive to Rebreathing) compared to the others (non-responsive to Rebreathing), indicating higher heart rate variability in these patients

**Fig. 2:**
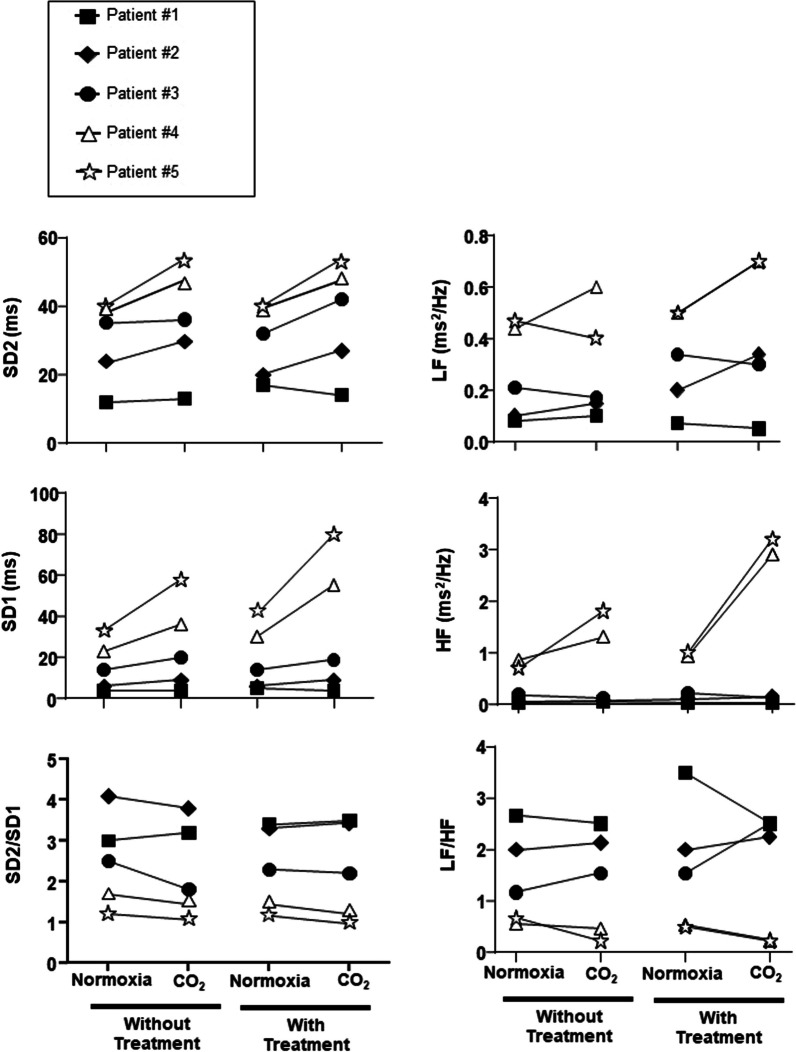
Poincaré indexes and Frequential parameters during and steady-state air breathing (normoxia) and CO_2_ challenge. Increases in vagal SD1 during hypercapnia were seen in Group 1 patients only, with or without (washout) treatment

Poincaré return maps (Fig. 1) in steady-state conditions indicated that, during Normoxia, Patients #1, #2 and #3 had a reduced HRV as compared to Patients #4 and #5, with lower SD1 and HF and higher SD2/SD1 and LF/HF, indicating an alteration of the autonomic nervous system in favour of an increase in sympathetic activity and reduced vagal function. This was true in the “without treatment” and the “with treatment” conditions (Fig. 2). During steady-state 5% hypercapnia, no apparent changes were seen in Patients #1, #2 and #3 whereas SD1 increased in Patients #4 and #5, both without treatment” and “with treatment” (Fig. 2).

## Discussion

In a very limited number of patients, this exploratory analysis may suggest that CCHS patients with PARM mutation who recover a ventilatory response to CO_2_ when prescribed desogestrel alone or levonorgestrel combined with ethinylestradiol had higher cardiac autonomic balance and some degree of cardiac response to CO_2_ at rest.

The current results also suggest that: (i) levonorgestrel, possibly combined with ethinylestradiol, may allow some patients to recover a ventilatory response to hypercapnia, as it was already suggested for desogestrel [4], (ii) all patients do not respond to these drugs and (iii) when present, recovery lasts several months after stopping the treatment. The two women of this study with a recovery of a ventilatory response to hypercapnia took the progestin for three to four years. However, one of the patients whose case was previously published [4] showed a response after only three weeks of treatment. It is therefore difficult to speculate on a possible role of treatment duration in the recovery of the ventilatory response to CO_2_.

Etonogestrel, a desogestrel precursor, has been shown to enhance the respiratory response to metabolic acidosis in newborn rats [15]. In addition, desogestrel activates c-FOS expression in raphe nuclei serotonergic cells [16]_._ Keeping in mind that serotonin released from the raphe nuclei to the nucleus tractus solitarius (NTS) facilitates vagal responses [17], and that Phox2B neurones present in the NTS may participate to central chemosensitivity [18], it could be postulated that the effects of desogestrel or levonorgestrel on hypercapnic responses and cardiac response to CO_2_ in Patients #4 and #5 depend on a preserved NTS serotonin responsiveness.

We acknowledge that further experiments with a higher number of patients are needed. Meanwhile, we submit that HRV analyses could help identify progestin-responder among CCHS patients.

## Data Availability

The datasets used and/or analyzed during the current study are available from the corresponding author and Pr Christian Straus on reasonable request.
